# Suppressing PKC-dependent membrane P2X3 receptor upregulation in dorsal root ganglia mediated electroacupuncture analgesia in rat painful diabetic neuropathy

**DOI:** 10.1007/s11302-018-9617-4

**Published:** 2018-08-07

**Authors:** Ya-feng Zhou, Xiao-ming Ying, Xiao-fen He, Sheng-Yun Shou, Jun-Jun Wei, Zhao-xia Tai, Xiao-mei Shao, Yi Liang, Fang Fang, Jian-qiao Fang, Yong-liang Jiang

**Affiliations:** 1 Department of Acupuncture, Zhejiang Provincial People’s Hospital, Hangzhou, 310014 China; 2 Zhejiang Chinese Medical University, Hangzhou, 310053 China; 3 Department of Massage, the Third Affliated Hospital of Zhejiang Chinese Medical University, Hangzhou, 310005 China; 4 Department of Neurobiology and Acupuncture Research, the Third Clinical Medical College, Zhejiang Chinese Medical University, Hangzhou, 310053 China

**Keywords:** Dorsal root ganglia (DRG), Electroacupuncture (EA), Painful diabetic neuropathy (PDN), Protein kinase C (PKC), P2X3 receptor

## Abstract

Painful diabetic neuropathy (PDN) is a common and troublesome diabetes complication. Protein kinase C (PKC)-mediated dorsal root ganglia (DRG) P2X3 receptor upregulation is one important mechanism underlying PDN. Accumulating evidence demonstrated that electroacupuncture (EA) at low frequency could effectively attenuate neuropathic pain. Our previous study showed that 2-Hz EA could relieve pain well in PDN. The study aimed to investigate whether 2-Hz EA relieves pain in PDN through suppressing PKC-mediated DRG P2X3 receptor upregulation. A 7-week feeding of high-fat and high-sugar diet plus a single injection of streptozotocin (STZ) in a dose of 35 mg/kg after a 5-week feeding of the diet successfully induced type 2 PDN in rats as revealed by the elevated body weight, fasting blood glucose, fasting insulin and insulin resistance, and the reduced paw withdrawal threshold (PWT), as well as the destructive ultrastructural change of sciatic nerve. DRG plasma membrane P2X3 receptor level and DRG PKC expression were elevated. Two-hertz EA failed to improve peripheral neuropathy; however, it reduced PWT, DRG plasma membrane P2X3 receptor level, and DRG PKC expression in PDN rats. Intraperitoneal administration of P2X3 receptor agonist αβ-meATP or PKC activator phorbol 12-myristate 13-acetate (PMA) blocked 2-Hz EA analgesia. Furthermore, PMA administration increased DRG plasma membrane P2X3 receptor level in PDN rats subject to 2-Hz EA treatment. These findings together indicated that the analgesic effect of EA in PDN is mediated by suppressing PKC-dependent membrane P2X3 upregulation in DRG. EA at low frequency is a valuable approach for PDN control.

## Introduction

Painful diabetic neuropathy (PDN) is a common and troublesome diabetes complication and occurs in ~ 20% of people with diabetes [1]. Patients with PDN often experience aberrant pain sensation, including spontaneous pain, hyperalgesia, and allodynia that seriously affect their life quality [2]. Studies show that several ion channels and receptors in dorsal root ganglia (DRG) are dysregulated in PDN, which make a contribution to sensitization of pain responses by enhancing excitability of nociceptor [3–6].

The P2X3 receptor is a subtype of the ionotropic receptors for adenosine 5′-triphosphate (ATP) and most highly expressed in pain-sensing sensory neurons of the DRG [7–9]. The P2X3 receptor is an important transducer of nociceptive stimuli and altered P2X3 receptor after nerve injury contributes to neuropathic pain hypersensitivity [10–13]. Upregulation of P2X3 receptor in DRG plays an important role in the development of PDN [4, 14, 15]. Protein kinase C (PKC) is an umbrella term for a family of serine/threonine kinases. Many studies which employed pharmacological methods have shown the important roles of the PKC family in the modulation of pain [16]. Hyperalgesia and C-fiber hyperexcitability to mechanical stimuli were reduced by inhibiting PKC in diabetic rats [17, 18]. It was found that P2X3 currents evoked by αβ-meATP in DRG neurons were suppressed by application of PKC inhibitor in inflammatory pain model [19]. However, whether the upregulation of DRG P2X3 receptor in PDN could be mediated by PKC has not yet been identified.

Although current medication for PDN is unsatisfactory or has significant side effects [3], the potential value of electroacupuncture, which is widely used in pain management nowadays, in treating PDN has begun to attract attention [20, 21]. Accumulating evidence demonstrated that electroacupuncture (EA) at low frequency could effectively attenuate neuropathic pain [22–25]. Consistent with this, our previous study showed that 2-Hz EA relieved pain in PDN rats well [21]. However, the exact mechanism underlying analgesia of EA at low frequency in PDN remains unclear. Previous studies showed that EA had downregulatory effects on PKC and P2X3 receptor in other animal models [26–28]. Thus, the present study aimed to investigate the effects of 2-Hz EA on DRG PKC and P2X3 receptor and whether PKC regulates P2X3 receptor, which may be involved in EA analgesia in PDN.

## Material and methods

### Animals

All animal studies were performed in accordance with the regulations of the State Science and Technology Commission for the care and use of laboratory animals (State Science and Technology Commission Order No. 2, 1988). Five-week-old male Sprague-Dawley rats were obtained from SLAC Laboratory Animal Co. Ltd., Shanghai, China, and were housed in Zhejiang Chinese Medical University (No. SYXK (zhe) 2013-0184). Rats were housed in temperature-controlled animal cages (25 ± 1 °C) under a 12-h light and 12-h dark cycle, with free access to food and water.

### Experimental design

Two experiments were conducted. Experiment 1 focused on the effects of 2-Hz EA on allodynia, DRG P2X3 receptor level, and PKC expression in type 2 PDN. Experiment 2 focused on the blocking effect of intraperitoneal administration of P2X3 receptor agonist αβ-meATP or PKC activator phorbol 12-myristate 13-acetate (PMA) on 2-Hz EA action.

### Establishment of type 2 PDN model and grouping

In experiment 1, 35 normal SD rats were randomly divided into the control group (*n* = 8, normal diet) and PDN modeling group (*n* = 27, the high-fat and high-sugar diet plus a single streptozotocin (STZ) injection). The formula of the high-fat and high-sugar diet was as follows: 72.5% normal diet plus 10% lard, 10% sucrose, 2% cholesterol, 0.5% sodium cholate, and 5% yolk powder. Body weight, fasting blood glucose (FBG), and fasting insulin (FINS) were measured at 0 and 5 W (5 weeks of feeding), and the insulin resistance (IR) was calculated accordingly. Rats in the PDN modeling group with IR were adopted after 5 weeks of feeding. These adopted rats were further given a single intraperitoneal injection of STZ (35 mg/kg, Sigma, St. Louis, MO, USA) after fasting for 12 h, with free access to water. Rats in the control group were injected the same dose of vehicle (citrate buffer). Body weight, FBG, FINS, and paw withdrawal threshold (PWT, to assess mechanical allodynia) were measured 2 weeks after STZ injection (7 W). Rats in the PDN modeling group with increased FINS level, IR, FBG ≧ 11.1 mmol/l, and PWT ≦ 85% base value were considered as successful type 2 PDN model rats (*n* = 16). These rats were then randomly divided into the PDN group and PDN + EA group (*n* = 8 per group).

The procedures for type 2 PDN establishment in experiment 2 were the same *as* those in experiment 1. The successful type 2 PDN rats were then randomly divided into the PDN + EA + PBS group, PDN + EA + αβ-meATP group, and PDN + EA + PMA group (*n* = 8 per group).

### FBG and body weight measurement

After 12 h of fasting with free access to water, tail vein blood was obtained. FBG was detected using a compact glucometer (Roche, China). After that, body weight was measured. FBG and body weight were measured on 0, 5 , and 7 W.

### Enzyme-linked immunosorbent assay for FINS and IR determination

After 12 h of fasting, but with free access to water, 1 ml of orbital venous blood was obtained. After standing at room temperature for 30 min, the blood samples were centrifuged at 3500× r/min for 10 min at 4 °C, and the serum samples were collected. FINS was measured using a rat insulin enzyme-linked immunosorbent assay (ELISA) kit (Cayman Chemical, USA) according to the manufacturer’s directions. The IR was then calculated using the following formula: HOMA-IR = (fasting glucose × fasting insulin) ∕ 22.5. Since the value is not normally distributed, the natural logarithm was used for the calculation. FINS and IR were assessed on 0, 5, and 7 W.

### EA treatment

Acupuncture needles of 0.25 mm in diameter were inserted approximately 5 mm deep into the bilateral acupoints Zusanli (ST36) and Kunlun (BL60). The ends of the needles in each side were attached to a pair of electrodes from an electrical stimulator (LH-202H, Huawei Co. Ltd., Beijing, China). EA (2 Hz, 0.4-ms pulse width) was administered for 30 min once daily for 7 consecutive days. The current was applied 1 mA for 15 min followed by 2 mA for another 15 min. Since the analgesic effects of the two acupoints are well documented [24, 29], we did not carry out sham acupuncture for control. Animals were awake and calmed by placing the heads in black hoods with no physical restraint during EA treatment. Rats were subject to the same calming procedure in the control and PDN groups.

### Administration of αβ-meATP and PMA

Rats in the PDN + EA + αβ-meATP group and the PDN + EA + PMA group were hypodermically injected with αβ-meATP (0.6 μmol/l, 100 μl) and PMA (0.2 ng/μl, 100 μl) into the ventral surface of each hind paw 10 min prior to EA treatment, respectively. Rats in the PDN + EA + PBS group received the same dose of vehicle (PBS buffer) as a control.

### Paw withdrawal threshold measurement

All tests were performed by an experimenter blinded to the treatment groups. PWT to a von Frey-like filament was measured to assess mechanical allodynia using a Dynamic Plantar Aesthesiometer (Ugo Basile, Italy) on 0, 5, and 7 W and at 30 min after EA treatment on day 1, 3, and 7 post 7 W. Rats were acclimatized in a clear plastic chamber for 30 min. A mechanical stimulus of a steel rod (0.5 mm in diameter) with ascending force (0–50 g) was pushed against the plantar surface of the hind paw until a strong and immediate withdrawal occurred. The stimulus was stopped automatically and the value was recorded as the PWT. Each hind paw PWT was calculated as the mean of three consecutive tests with intervals of 5 min. Rat PWT was determined as (PWT of left hind paw + PWT of right hind paw) ∕ 2.

### Electron microscopy study on sciatic nerve

After deep anesthesia with chloral hydrate (350 mg/kg, i.p.), rats were perfused transcardially with ice-cold saline. The central segments of bilateral sciatic nerves with a distance of 2 mm to the proximal end and 3 mm to the distal end were dissected and placed in the fixative solution (2.5% glutaraldehyde in 0.1 M sodium cacodylate buffer, pH 7.0) at 4 °C overnight. The tissues were rinsed in phosphate-buffered saline (0.1 M, pH 7.0) for three times with 15 min for each time, post-fixed in 1% osmic acid for 2 h, rinsed again in phosphate-buffered saline (0.1 M, pH 7.0) for three times with 15 min for each time, dehydrated in ascending concentrations of ethanol solutions (50–100%), and embedded in Spurr resin. Ultrathin sections (70 nm) were cut with an ultramicrotome (Leica, UC7, Germany). Sections were then stained with uranyl acetate and lead citrate and observed with a Hitachi H-7650 transmission electron microscope (Hitachi, H-7650, Japan).

### Western blotting analysis of DRG total and plasma membrane P2X3 receptor

After deep anesthesia with chloral hydrate (350 mg/kg, i.p.), L4–L6 DRGs were collected and rapidly frozen in liquid nitrogen. Total protein was collected as follows: bilateral L4, L5, and L6 DRGs were sonicated on ice in RIPA Lysis Buffer (Beyotime, China) with an addition of protease inhibitor cocktail (Sangon Biotech, China), centrifuged at 10,000× rpm for 10 min at 4 °C, and then the supernatants were collected.

Plasma membrane protein of L4, L5, and L6 DRGs was extracted using Membrane and Cytosol Protein Extraction Kit (Beyotime, China) according to manufacturer’s instructions. Protein concentration was determined by the bicinchoninic acid method. Protein extracts of rats in each group were equally pooled according to their concentrations. Samples were mixed with an equal volume of 2× sample loading buffer and denatured by boiling at 100 °C for 5 min. Proteins (30 μg/lane) were separated by an 10% SDS PAGE gel for P2X3 receptor and GAPDH and then transferred to polyvinylidene difluoride membranes (Bio-Rad, USA). After being blocked in 0.01 M TBS with 0.1% Tween 20 and 5% dehydrated skim milk, the membranes were incubated with rabbit anti-rat P2X3 (1:1000; Abcam, USA) and rabbit anti-rat GAPDH (1:1000; Abcam, USA) overnight at 4 °C. After being washed, the membrane for GAPDH was visualized by chemiluminescence (ECL Plus; Amersham, USA), while the membrane for P2X3 receptor was incubated with the species-specific secondary antibody for 1 h at room temperature and then washed and visualized by chemiluminescence (ECL Plus; Amersham, USA). Bands were detected by an Image Quant LAS 4000 system (Fujifilm, Japan) with Image Quant TL 7.0 software (GE Healthcare, USA). Target protein levels were normalized against GAPDH levels and then expressed as relative fold changes compared to the control group in experiment 1 and the PDN + EA + PBS group in experiment 2 [30, 31]. Five rats of each group were analyzed.

### Immunofluorescence study of DRG *p-PKCε*

After deep anesthesia with chloral hydrate (350 mg/kg, i.p.), rats were perfused transcardially with 150-ml ice-cold sterilized saline followed by 500 ml cold, fresh 4% paraformaldehyde in 0.1 M phosphate-buffered saline (PBS, pH 7.4). Bilateral L4–L6 DRGs were dissected, post-fixed in the same fixative for 3 h, and then consecutively immersed in 15% (*w*/*v*) and 30% (*w*/*v*) sucrose solution overnight at 4 °C. DRGs were embedded in OCT (Bayer Corp, Elkhart, IN), frozen and then cut into 14-μm sections. Sections were mounted on glass slides, rinsed in PBS, and blocked in PBS containing 10% goat serum for 1 h at 37 °C. Then, sections were incubated with rabbit anti-rat *p-PKCε* (1:2000; Abcam, USA) which was dissolved in PBS containing 10% goat serum for 14 h at 4 °C. After being washed in PBS, sections were incubated with Alexa Fluor594-conjugated goat anti-rabbit IgG (1:800; Jackson Immunoresearch, USA) for 1 h at 37 °C. After immunostaining, sections were rinsed in PBS and coverslipped with mounting medium. Images were obtained using a fluorescence microscope (Nikon A1R; Nikon, Japan) equipped with NIS-Elements AR 3.2 64-bit software. Immunoreactivity was quantified using Image-Pro Plus 6.0 software (Thermo, USA) under blinded conditions. The ratio of positive immunoreactivity was determined as the percentage of positive DRG neurons in the total DRG neurons. Five non-consecutive sections were calculated for the average of each rat, and three rats were analyzed for each group.

### Statistical analysis

The data were expressed as mean ± standard deviation (SD). Independent sample *t* test was used for comparison between two groups. Statistical analysis for three groups was performed by one-way analysis of variance (ANOVA) followed by post hoc test of the least significant difference (LSD) for multiple comparisons. *P* < 0.05 was considered statistically significant.

## Results

### Rat type 2 PDN was successfully induced by the high-fat and high-sugar diet plus a single small dose injection of STZ

**Fig. 1:**
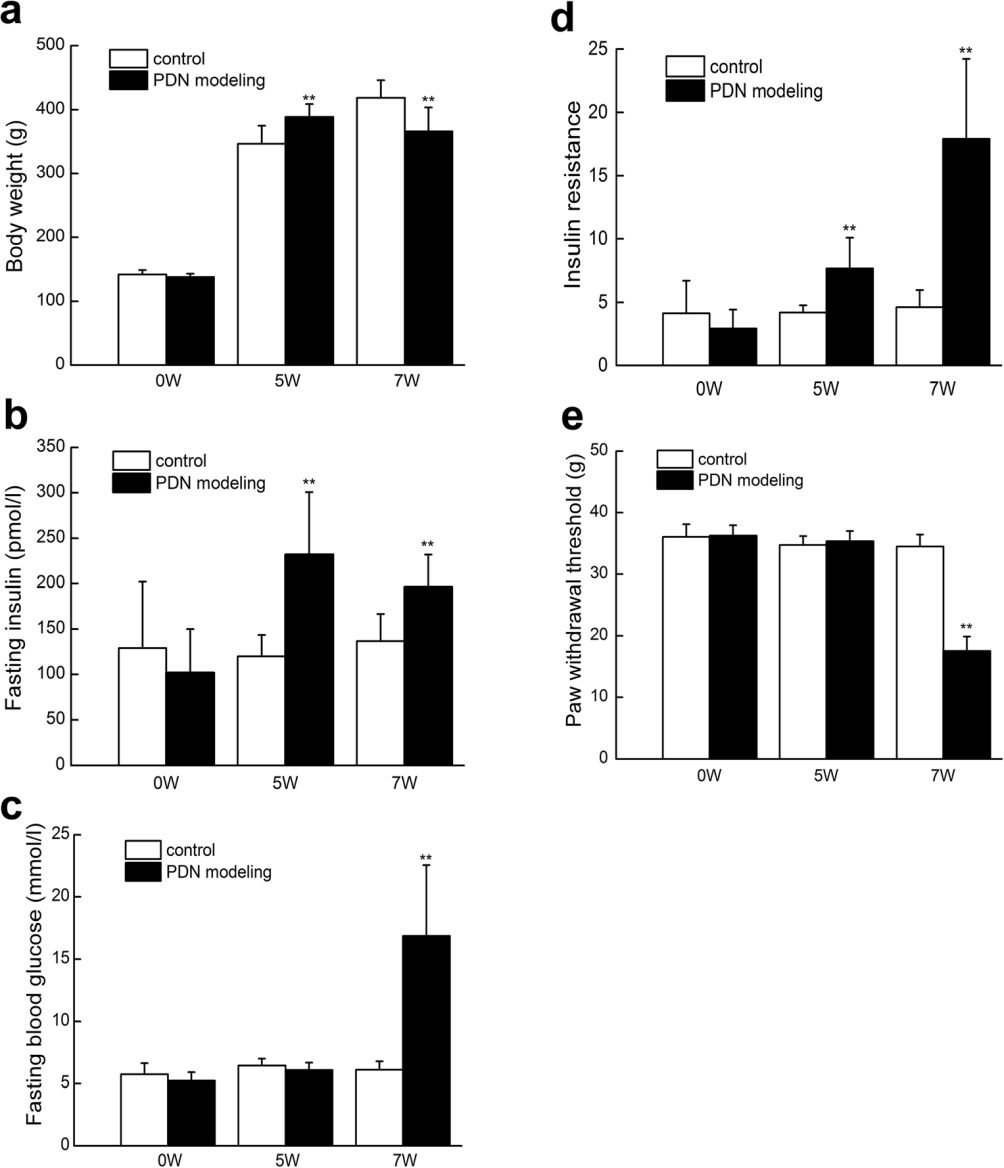
**a**–**e** Changes in body weight, fasting insulin, fasting blood glucose, insulin resistance, and paw withdrawal threshold of rats subjected to a high-fat, high-sugar diet for 7 weeks and a single intraperitoneal injection of STZ in a dose of 35 mg/kg at 5 W. Data were presented as the mean ± SD and analyzed using independent sample *t* test. *n* = 8 for the control group and *n* = 16 for the ***PDN*** modeling group. ^∗∗^*P* < 0.01, compared with the control group

After being fed with the diet composed of 72.5% normal diet, 10% lard, 10% sucrose, 2% cholesterol, 0.5% sodium cholate, and 5% yolk powder for 5 weeks, rats’ body weight, FINS, and IR were significantly increased as compared to those in the control group (*P* < 0.01, respectively) (Fig. 1a–d). After a single intraperitoneal injection of STZ in a dose of 35 mg/kg at 5 W and additional 2 weeks of high-fat and high-sugar diet, a significant increase in FBG (Fig.1c) and a decrease in PWT were found (*P* < 0.01, respectively) (Fig.1e). The increased FINS and IR levels were still sustained (*P* < 0.01, respectively). In the PDN modeling group, 16 in 27 rats successfully developed type 2 PDN.

### Two-hertz EA relived mechanical allodynia in PDN

**Fig. 2:**
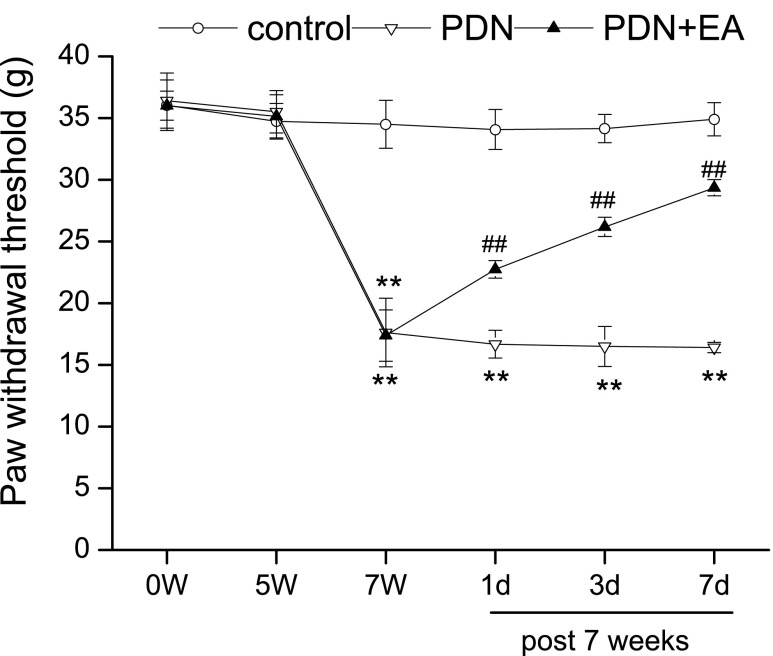
Effect of 2-Hz EA on paw withdrawal threshold (PWT) of PDN rats. EA treatment was administered once every day for 7 consecutive days from 7 W. Data were presented as mean ± SD, *n* = 8 per group. ^∗∗^ *P* < 0.01, compared with the control group; ^##^ *P* < 0.01, compared with the PDN group

Rats in the PDN, PDN + 2-Hz EA groups developed mechanical allodynia in bilateral hind paws, as shown by the significant reductions of PWTs when compared to that in the control group on 7 W (Fig. 2; *P* < 0.01, respectively). Mechanical allodynia was sustained in the PDN group until the end of experiment. Two-hertz EA significantly increased PDN rat PWT as compared to that of PDN controlled rat (*P* < 0.01, respectively).

### Two-hertz EA did not improve diabetic peripheral neuropathy

**Fig. 3:**
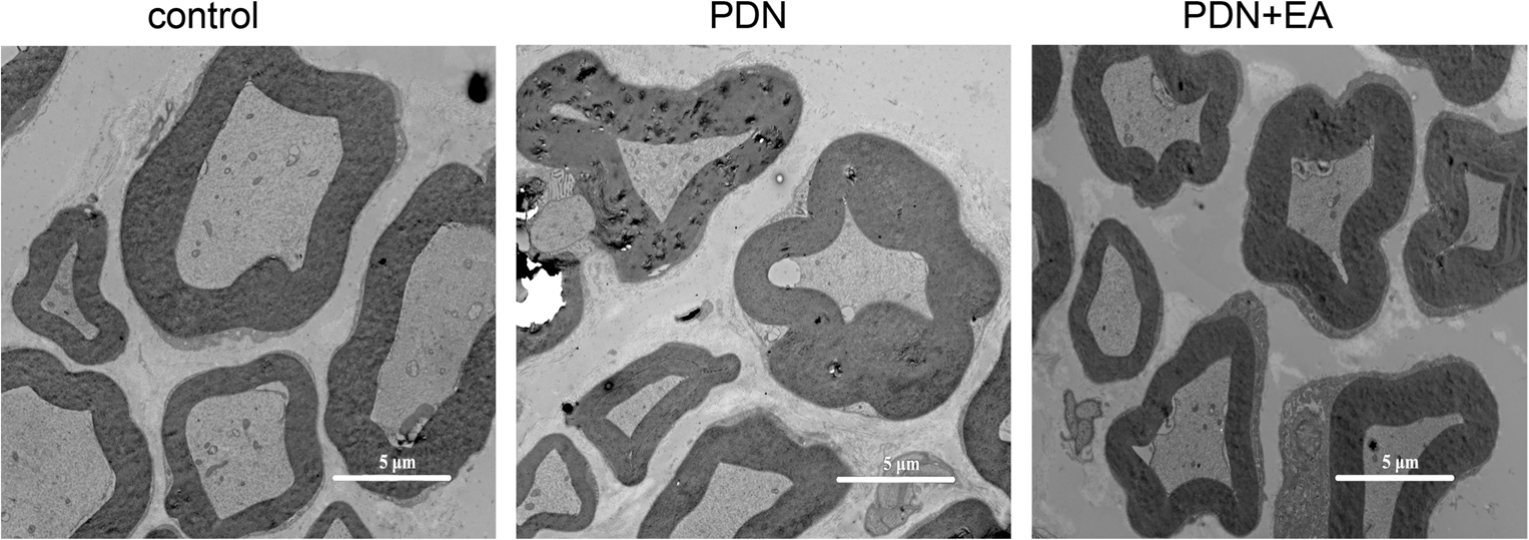
Ultrastructural changes of sciatic nerve by electron microscopy. × 8000 magnification (scale bar = 5 μm)

Transmission electron microscope study confirmed diabetic peripheral neuropathy. Myelin disruption and dissolute axoplasm of sciatic nerve fiber were found in PDN rats. No obvious change in histomorphology of sciatic nerve was found between the PDN group and PDN + EA group (Fig. 3).

### Two-hertz EA suppressed upregulation of blood membrane protein levels of P2X3 receptor in L4–L6 DRGs of PDN rats

**Fig. 4:**
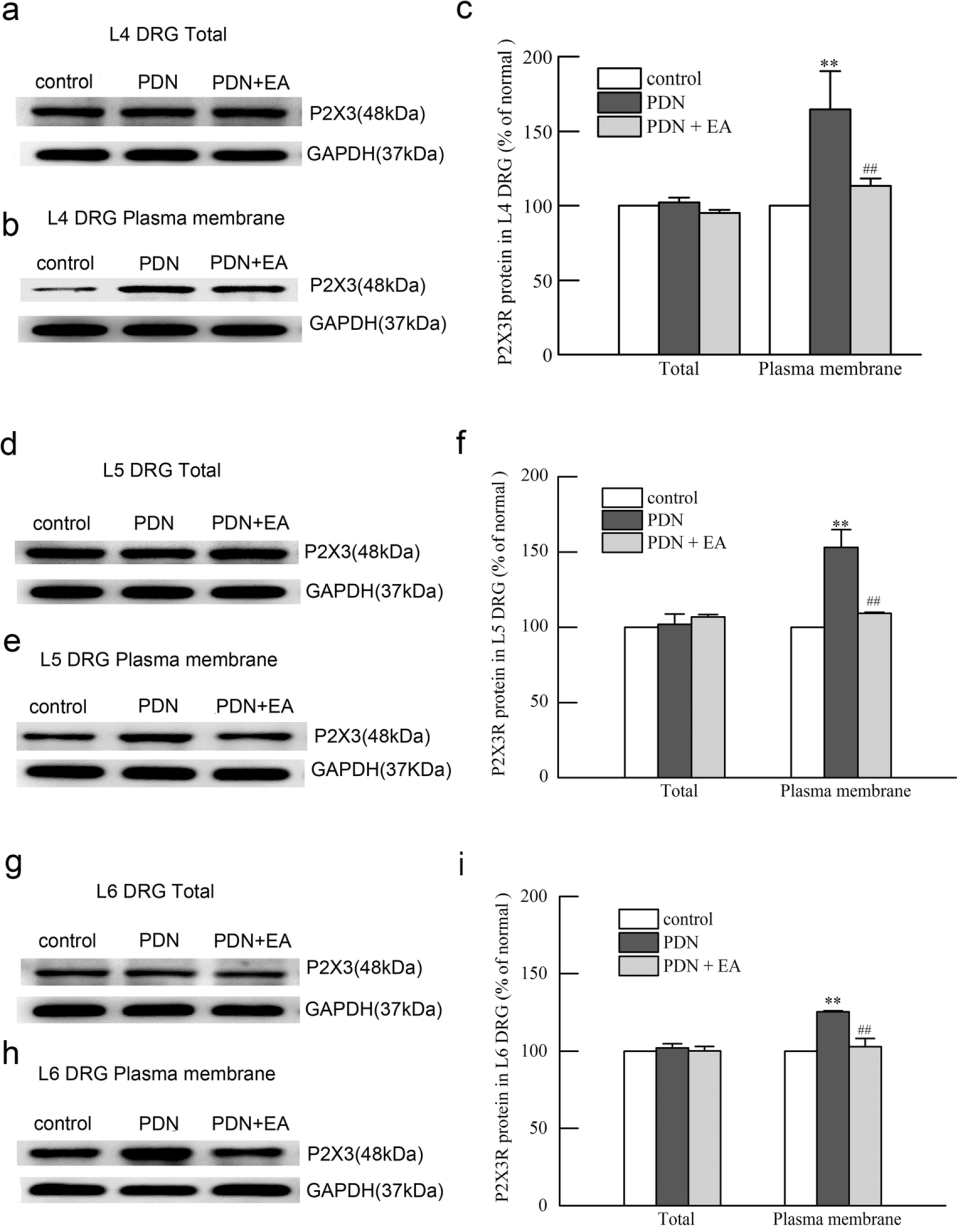
Effects of 2-Hz EA on total and plasma membrane protein levels of P2X3 receptor in L4, L5, and L6 DRGs of PDN rats after EA treatment for 7 days. **a**, **b** Representative Western blot protein and **c** relative amounts of total and plasma membrane P2X3 receptor in L4 DRGs. **d**, **e** Representative Western blot protein and **f** relative amounts of total and plasma membrane P2X3 receptor in L5 DRGs. **g**, **h** Representative Western blot protein and **i** relative amounts of total and plasma membrane P2X3 receptor in L6 DRGs. Results were expressed as relative fold changes as compared to the control group after normalization to GAPDH. Data were presented as mean ± SD of three independent experiments, *n* = 5 per group. ^∗∗^ *P* < 0.01, compared with the control group; ^##^*P* < 0.01, compared with the PDN group

No significant change of total protein levels of P2X3 receptor in L4–L6 DRGs was found between the PDN group and the control group and between the PDN + EA group and the PDN group (*P* > 0.01, respectively) (Fig. 4). The plasma membrane protein levels of P2X3 receptor in L4–L6 DRGs were significantly elevated in the PDN group as compared to those in the control group (*P* < 0.01, respectively) (Fig. 4), which were significantly suppressed by 2-Hz EA when compared to PDN controlled rats (*P* < 0.01, respectively).

### Two-hertz EA suppressed upregulation of p-PKC expressions in L4–L6 DRGs of PDN rats

**Fig. 5:**
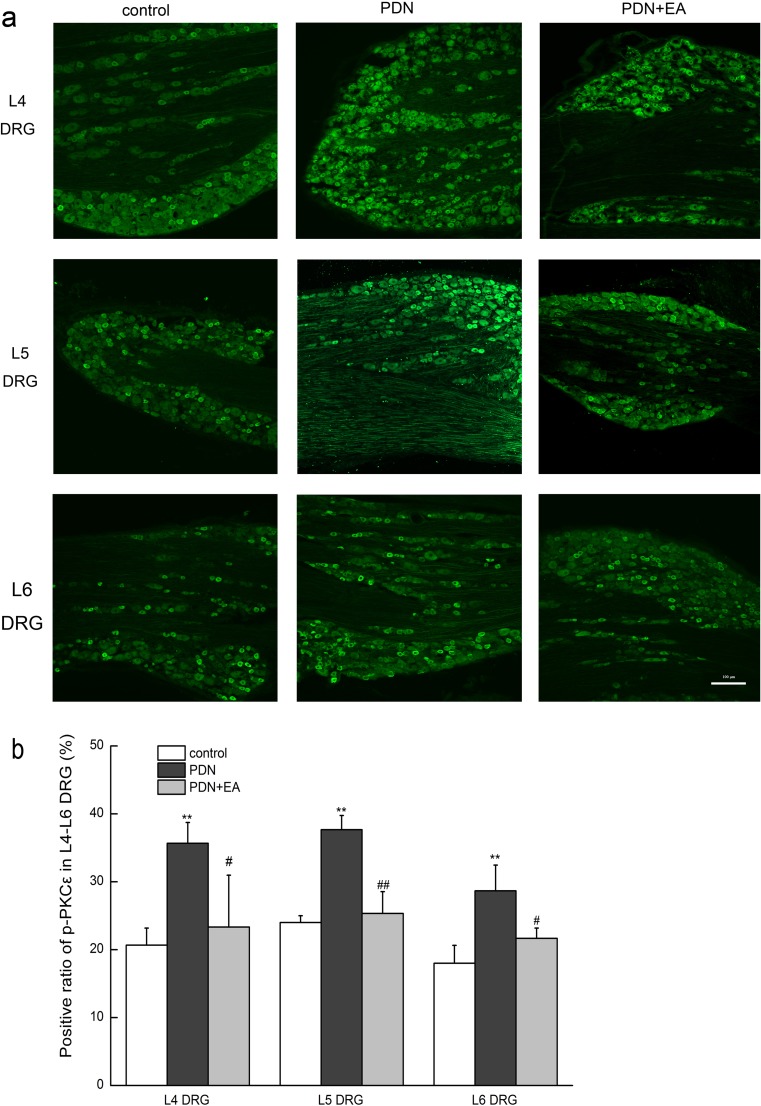
Effects of 2-Hz EA on p-PKC***ε*** expressions in L4–L6 DRGs of PDN rats after EA treatment for 7 days. **a** Representative bright-field micrographs showing p-PKC***ε***-immunoreactive neurons in L4, L5, and L6 DRGs of rats. Scale bar = 100 μm. **b** Statistical analysis of L4, L5, and L6 DRG p-PKC***ε***-immunoreactive neurons. Scale bar = 100 μm. Data were presented as mean ± SD, *n* = 3 per group. ^∗∗^*P* < 0.01, compared with the control group; ^#^*P* < 0.05, ^##^*P* < 0.01, compared with the PDN group

The p-PKC-immunoreactive cells were mainly small-to-medium DRG neurons (20–50 μm). The expressions of p-PKC in L4–L6 DRGs were significantly elevated in the PDN group as compared to those in the control group (푃 < 0.01, respectively) (Fig. 5), which were significantly suppressed by 2-Hz EA when compared to PDN controlled rats (푃 < 0.05 for L4 and L6 DRGs, *P* < 0.01 for L5 DRG).

### Intraperitoneal injection of αβ-meATP or PMA blocked 2-Hz EA analgesia

**Fig. 6:**
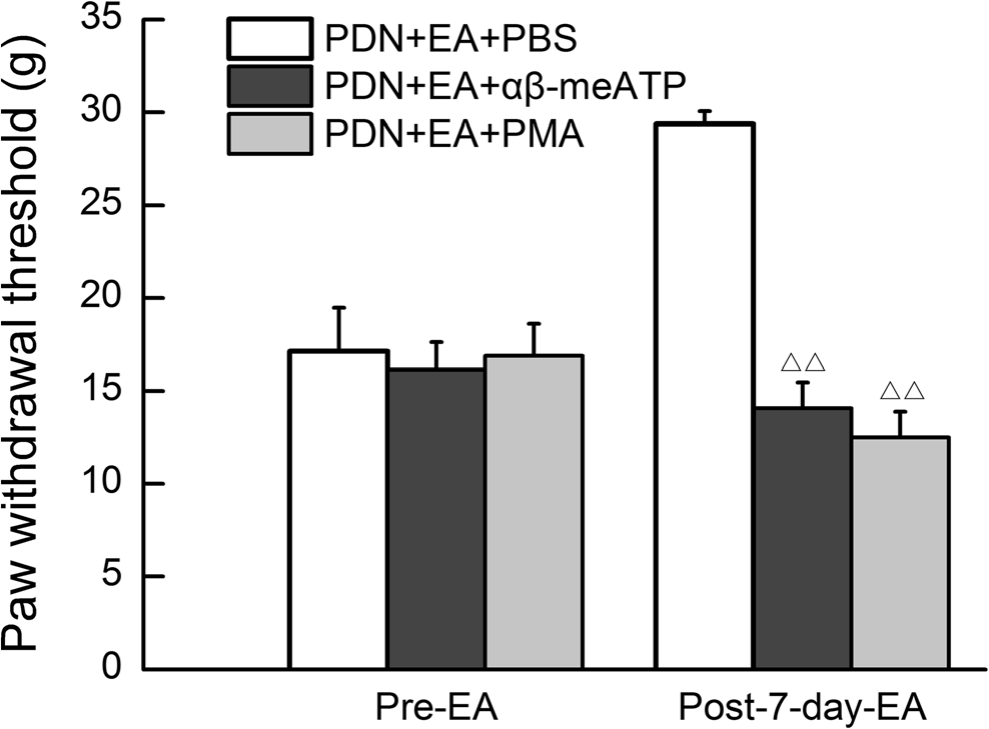
Effect of intraperitoneal injection of αβ-meATP or PMA on 2-Hz EA analgesia in PDN rats as revealed by paw withdrawal threshold (PWT). EA treatment was administered once every day for 7 consecutive days after establishment of PDN model. Rats in the PDN + EA + αβ-meATP group and the PDN + EA + PMA group were hypodermically injected with αβ-meATP (0.6 μmol/l, 100 μl) and PMA (0.2 ng/μl, 100 μl) into the ventral surface of hind paws 10 min prior to EA treatment respectively. Rats in the PDN + EA + PBS group received the same dose of PBS buffer as a control. Data were presented as mean ± SD, *n* = 8 per group. ^△△^ *P* < 0.01, compared with the PDN + EA + PBS group

As shown in Fig. 6, the intraperitoneal injection of αβ-meATP or PMA significantly blocked the analgesic effect of 2-Hz EA on PDN rats as compared to the PBS injection controlled group (*P* < 0.01, respectively).

### Intraperitoneal injection of PMA reversed downregulation of the plasma membrane protein levels of P2X3 receptor in L4–L6 DRGs of PDN rat by 2-Hz EA

**Fig. 7:**
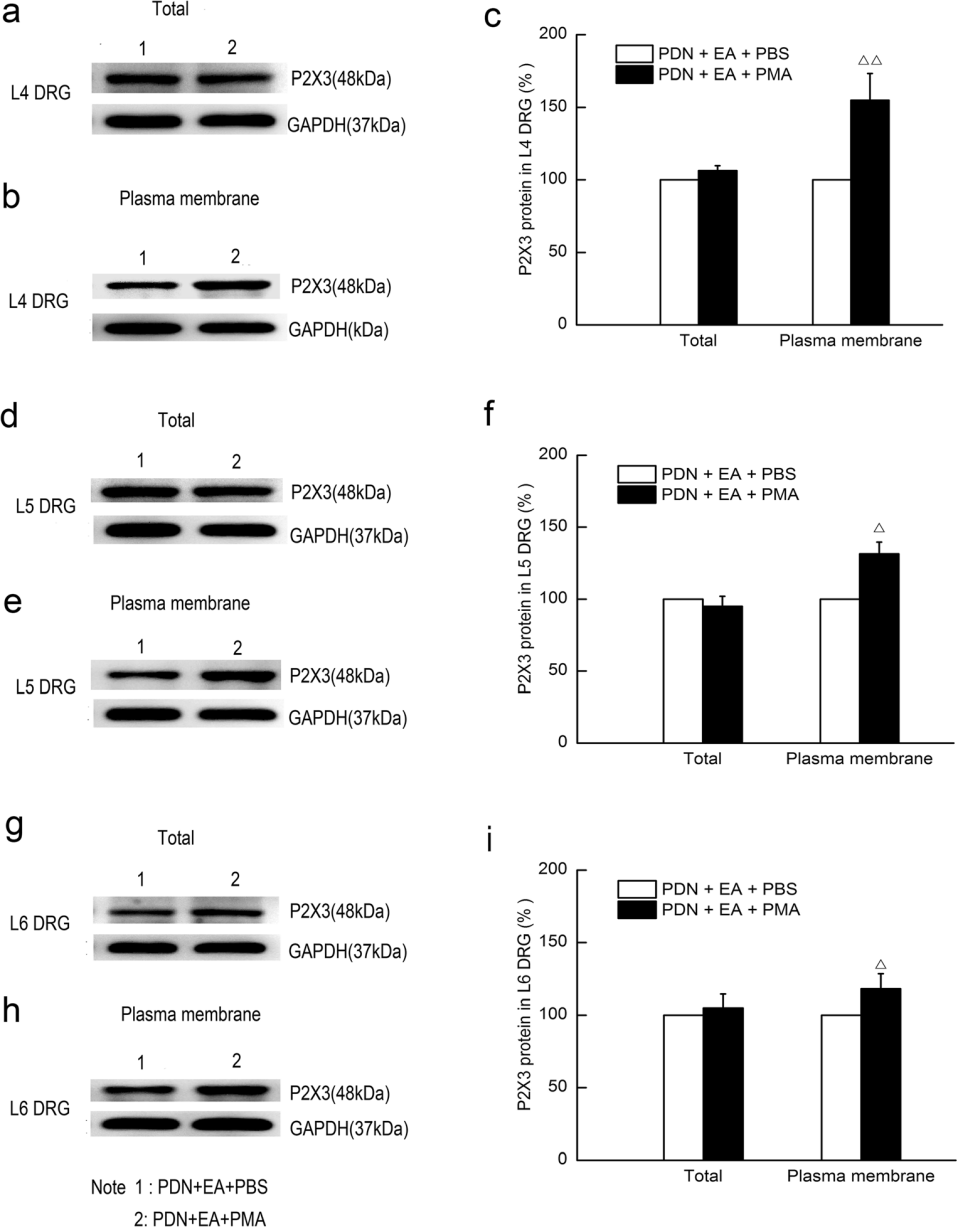
Effects of intraperitoneal injection of PMA on total and plasma membrane protein levels of P2X3 receptor in L4, L5, and L6 DRGs of PDN rats subject to 2-Hz EA after EA treatment for 7 days. PMA was intraperitoneally injected (0.2 ng/μl, 100 μl) into the ventral surface of hind paw 10 min prior to EA treatment. Rats in the PDN + EA + PBS group received the same dose of PBS buffer as a control. **a**, **b** Representative Western blot protein and **c** relative amounts of total and plasma membrane P2X3 receptor in L4 DRGs. **d**, **e** Representative Western blot protein and **f** relative amounts of total and plasma membrane P2X3 receptor in L5 DRGs. **g**, **h** Representative Western blot protein and **i** relative amounts of total and plasma membrane P2X3 receptor in L6 DRGs. Results were expressed as relative fold changes as compared to the PDN + EA + PBS group after normalization to GAPDH. Data were presented as mean ± SD of three independent experiments, *n* = 5 per group. ^△^ *P* < 0.05, ^△△^ *P* < 0.01, compared with the PDN + EA + PBS group

No significant difference in the total protein levels of P2X3 receptor in L4–L6 DRGs was found between the PDN + EA + PBS group and the PDN + EA + PMA groups (Fig. 7). Intraperitoneal injection of PMA significantly increased the plasma membrane protein levels of P2X3 receptor in L4–L6 DRGs of PDN rat subject to 2-Hz EA treatment as compared to the PBS injection controlled group (*P* < 0.01, respectively).

## Discussion

The present study investigated whether 2-Hz EA exerted analgesic effect on PDN rats by suppressing DRG P2X3 receptor through PKC pathway. We found that a long duration of high-fat and high-sugar diet plus a single intraperitoneal injection of STZ in a small dose induced type 2 PDN in rats. It caused peripheral neuropathy, mechanical allodynia, and elevated DRG plasma membrane P2X3 receptor level and DRG PKC expression. Although 2-Hz EA did not effectively improve peripheral neuropathy, it attenuated mechanical allodynia and downregulated DRG plasma membrane P2X3 receptor level and DRG PKC expression. Intraperitoneal administration of αβ-meATP or PMA blocked 2-Hz EA analgesia. Furthermore, PMA administration also increased DRG plasma membrane P2X3 receptor level in PDN rats subject to 2-Hz EA treatment. These findings together indicated that suppressing PKC-mediated DRG plasma membrane P2X3 receptor upregulation played an important role in 2-Hz EA analgesia in PDN.

The current experimental models of PDN are usually made by a single large injection of STZ due to the convenient induction of hyperglycemia [32–34]. Due to the selective destruction of pancreatic beta cells, these models are characteristic of rapid onset, weight loss, and insulin deficiency, which can well represent type 1 diabetes, but fail to mimic the pathological process of type 2 diabetes, the predominant subtype of diabetes nowadays, like insulin resistance. Actually, owing to the different durations, PDN is of more great significance to type 2 than to type 1 diabetes. Thus, it is of great value to study PDN under a condition of type 2 diabetes. In the present study, a 7-week feeding of a high-fat and high-sugar diet plus a single injection of STZ in a dose of 35 mg/kg at 5 W successfully induced type 2 PDN in rats as revealed by the elevated body weight, FBG, FINS, IR, and the reduced PWT, as well as the destructive changes of sciatic nerve.

P2X3 receptor is a ligand-gated ion channel and plays a crucial role in facilitating pain transmission at the peripheral and spinal sites in neuropathic pain [8, 35]. Studies showed that PDN was closely related to the enhancement of DRG P2X3 receptor function and expression [4, 14]. P2X3-mediated currents in DRG neurons and the expression of DRG plasma membrane P2X3 receptor were all enhanced in STZ-induced PDN rats [4]. Meanwhile, P2X3 antagonist PPADS or TNP-ATP could inhibit STZ-induced mechanical allodynia [4, 14]. Interference in P2X3 receptor gene expression by siRNA could reduce mechanical allodynia induced by αβ-meATP in rats [15]. Our results were consistent with these findings. We found that the level of DRG plasma membrane P2X3 receptor was increased in type 2 PDN rats. Although 2-Hz EA did not effectively improve diabetic peripheral neuropathy, it attenuated mechanical allodynia and downregulated DRG plasma membrane P2X3 receptor level. Furthermore, 2-Hz EA analgesia was blocked by administration of αβ-meATP. These results together suggested that 2-Hz EA relieved neuropathic pain in PDN by downregulating DRG plasma membrane P2X3 receptor level.

Actually, allodynia caused by PDN could be relieved either through alleviating the damage to myelin structure or affecting sensory neuron sensitivity [36]. Koumine, a Benth alkaloid, showed anti-allodynic and neuroprotective effects in a large dose, while in a small dose, it just attenuated mechanical allodynia but failed to improve sciatic nerve histological change in PDN [36]. In the current study, EA only had an effect on DRG plasma membrane P2X3 receptor. It seemed that EA was not strong enough to produce a neuroprotective effect for PDN rats. EA analgesia was probably mainly accomplished through altering pain modulation-related receptors or signaling pathway.

PKC is widely involved in diabetic complications including PDN [37, 38]. It was reported that PKC inhibitor suppressed P2X3-mediated currents, while its activator potentiated P2X3-mediated currents, and induced trafficking of P2X3 receptor from the cytoplasm to the plasma membrane by activating protease-activated receptor-2 [19]. In the present study, we found that the expression of DRG PKC was enhanced, which was suppressed by 2-Hz EA. Administration of PMA blocked the analgesia of 2-Hz EA and increased the level of DRG plasma membrane P2X3 receptor. These results together indicated that 2-Hz EA may suppress DRG PKC and subsequently downregulate DRG plasma membrane P2X3 receptor, which contributed to its analgesia in PDN rats.

In conclusion, this is the first report demonstrating that the analgesic effect of EA in PDN is mediated by a downregulation of PKC-dependent P2X3 signaling in DRG. Our study provided scientific evidence for the application of EA at low frequency in PDN control.

## Abbreviations

DRG: Dorsal root ganglia
EA: Electroacupuncture
PMA: Phorbol 12-myristate 13-acetate
ELISA: Enzyme-linked immunosorbent assay
FBG: Fasting blood glucose
FINS: Fasting insulin
IR: Insulin resistance
PBS: Phosphate-buffered saline
PDN: Painful diabetic neuropathy
PKC: Protein kinase C
PWT: Paw withdrawal threshold
STZ: Streptozotocin
